# ADAMTS4 and ADAMTS5 Knockout Mice Are Protected from Versican but Not Aggrecan or Brevican Proteolysis during Spinal Cord Injury

**DOI:** 10.1155/2014/693746

**Published:** 2014-07-03

**Authors:** Kadir Demircan, Vehap Topcu, Tomoyuki Takigawa, Sumeyya Akyol, Tomoko Yonezawa, Gulfer Ozturk, Veli Ugurcu, Rukiye Hasgul, M. Ramazan Yigitoglu, Omer Akyol, Daniel R. McCulloch, Satoshi Hirohata

**Affiliations:** ^1^Department of Medical Biology, Turgut Ozal University School of Medicine, 06200 Ankara, Turkey; ^2^Department of Medical Genetics, Zekai Tahir Burak Women's Health Training and Research Hospital, 06230 Ankara, Turkey; ^3^Department of Molecular Biology and Biochemistry, Okayama University Graduate School of Medicine, Dentistry and Pharmaceutical Sciences, 2-5-1 Shikata-cho, Kita-ku, Okayama 700-8558, Japan; ^4^Vocational School of Health Sciences, Turgut Ozal University, 06374 Ankara, Turkey; ^5^Department of Biochemistry, Gaziosmanpasa University School of Medicine, 60000 Tokat, Turkey; ^6^Department of Clinical Biochemistry, Diskapi Yildirim Beyazit Training and Research Hospital, 06200 Ankara, Turkey; ^7^Private Bilecik Orhangazi Dialysis Center, 11000 Bilecik, Turkey; ^8^Department of Biochemistry, Turgut Ozal University School of Medicine, 06200 Ankara, Turkey; ^9^Department of Biochemistry, Hacettepe University Medical Faculty, 06200 Ankara, Turkey; ^10^School of Medicine, Faculty of Health, Deakin University, 75 Pigdons Road, Waurn Ponds, VIC 3216, Australia; ^11^Molecular and Medical Research SRC, Deakin University, 75 Pigdons Road,Waurn Ponds, VIC 3216, Australia

## Abstract

The chondroitin sulfate proteoglycans (CSPGs) aggrecan, versican, and brevican are large aggregating extracellular matrix molecules that inhibit axonal growth of the mature central nervous system (CNS). ADAMTS proteoglycanases, including ADAMTS4 and ADAMTS5, degrade CSPGs, representing potential targets for ameliorating axonal growth-inhibition by CSPG accumulation after CNS injury. We investigated the proteolysis of CSPGs in mice homozygous for *Adamts4* or *Adamts5* null alleles after spinal cord injury (SCI). ADAMTS-derived 50–60 kDa aggrecan and 50 kDa brevican fragments were observed in *Adamts4−/−*, *Adamts5−/−*, and *wt* mice but not in the sham-operated group. By contrast *Adamts4−/−* and *Adamts5−/−* mice were both protected from versican proteolysis with an ADAMTS-generated 70 kDa versican fragment predominately observed in WT mice. ADAMTS1, ADAMTS9, and ADAMTS15 were detected by Western blot in *Adamts4−/−* mice' spinal cords after SCI. Immunohistochemistry showed astrocyte accumulation at the injury site. These data indicate that aggrecan and brevican proteolysis is compensated in *Adamts4−/−* or *Adamts5−/−* mice by ADAMTS proteoglycanase family members but a threshold of versican proteolysis is sensitive to the loss of a single ADAMTS proteoglycanase during SCI. We show robust ADAMTS activity after SCI and exemplify the requirement for collective proteolysis for effective CSPG clearance during SCI.

## 1. Introduction

The extracellular matrix (ECM) is important for structural and functional development and maintenance of the central nervous system (CNS). The hyalectan (hyaluronan and lectin) binding class of chondroitin sulfate proteoglycans (CSPGs) comprises aggrecan, versican, neurocan, and brevican, each sharing a common N-terminal G1 domain invariably linked to hyaluronan, and a G3 C-terminal domain [1, 2]. Large O-linked glycosaminoglycan (GAG) chains attached to serine or threonine residues modify several splice variants of versican, aggrecan, brevican, and neurocan. Chondroitin sulphate moieties covalently attached to GAG chains confer a high negative charge attracting water into the tissue, providing rigidity and structure. Digestion of GAG chains using chondroitinase ABC creates a permissive media for axonal regrowth, in turn promoting regain of function. On the other hand, the loss of core proteins after chondroitinase ABC digestion maintains an inhibitory habitat at the injury site [1].

Adult mammalian CNS has poor plasticity to accomplish proper axon regeneration and sprouting. Glial scar formation caused by the reactive astrocyte response after traumatic injuries in the CNS is a major impediment to axonal regeneration partly due to CSPG production that is inhibitory for axonal regeneration [1]. Versican is predominately localized to nodes of Ranvier whilst aggrecan, brevican, and neurocan are present in axonal coats [3–6]. Recently, aggrecan, versican, and brevican were shown to inhibit axonal regeneration and myelination after neuronal injury [4–6]. These observations drew focus to the possibility of promoting proteolysis of hyalectans to favor gain-of-function after spinal cord injury.

ADAMTS4 and ADAMTS5 (aggrecanase-1 and aggrecanase-2, resp.) are the predominant enzymes in cartilage breakdown [7, 8], although recent studies revealed significant aggrecanolysis in articular cartilage of ADAMTS4 or ADAMTS5 knockout mice suggesting additional aggrecanase activity by one or more members of the ADAMTS aggrecanase family yet to be identified in the arthritic joint [9, 10]. Previous studies identified roles for ADAMTS4 and ADAMTS5 in spinal cord pathology; however, their role during spinal cord injury remains unclear. Tauchi et al. [6] showed that ADAMTS4 promoted functional recovery after SCI by cleaving CSPGs. We recently showed that* Adamts1*,* Adamts5,* and* Adamts9* mRNA expression were increased in mice following SCI [5]; however, the significance of this observation was not explored.

The biological importance of versican, a widely expressed transitional matrix PG [11], and its cleavage by ADAMTS aggrecanases is exemplified during developmental morphogenesis and reproduction. Despite ADAMTS5 single knockout mice being previously reported as phenotypically normal [7, 8, 12], those mice present with limb and heart abnormalities [13–15] where a reduction or absence of versican processing is also observed. An ADAMTS-generated N-terminal versican fragment rescued the ADAMTS-deficient limb-defect phenotype showing unequivocally that versican processing was required for developmental morphogenesis of the distal limb [15]. In ADAMTS1 knockout mice reduced or absent versican processing is observed in ovarian follicles and those mice have significantly lower fertility rates than wild-type mice due to anovulation [16].

The fact that ADAMTS proteoglycanases confer their substrate specificity towards the hyalectan class of CSPGs makes them ideal candidate therapeutic targets to pursue in SCI. The present study focused on ADAMTS4 and ADAMTS5 because of their previously highlighted roles in aggrecan and versican processing in contexts such as arthritis progression and developmental morphogenesis. Here, we report the proteolysis of three major substrates for these enzymes in spinal cords of* Adamts4*−/− or* Adamts5*−/− mice after spinal cord injury.

## 2. Material and Methods

### 2.1. Animals Experiments

Animal procedures were approved by the Okayama University, Okayama, Japan Animal Ethics Committee, in accordance with the International Care and Use of Animals in Research guidelines.* Adamts4* (*B6.129P2*-Adamts4^tm1Dgen^/*J*) and* Adamts5* (*B6.129P2*-Adamts5^tm1Dgen^/*J*) knockout mice were obtained from The Jackson Laboratory and are previously described [15, 17]. Spinal cord injury was induced in mice as previously described [5]. Briefly, mice were anesthetized with an I.P. injection of 50 mg/kg pentobarbital. Mice underwent a single level laminectomy and the dura was exposed at the middle thoracic level. A 3 g weight was dropped from 25 mm height using a modified NYU impactor to produce moderate contusion (1.3 mm) injury. This procedure was performed in three groups of 7 mice, group 1: wild-type, group 2:* Adamts4−/−,* and group 3:* Adamts5−/−*. Sham-operated (no injury) mice were also used as control (*n* = 7). For the sham group, only laminectomy was performed and surgical sites were sutured in layers.

### 2.2. Protein Analysis

Central lesion points in spinal cords were dissected 3 mm in length. Protein was extracted using a commercial kit (Cell lytic M Cell, sigma, St. Louis, MO). Western blot analysis was performed on days 1 (aggrecan and brevican) and 7 (versican and brevican) to examine their degradation products in all experimental groups. Protein concentration was determined using a Bradford assay kit (Biorad). Ten *μ*g of total protein per experimental group was boiled and electrophoresed on 4–15% sodium dodecyl sulfate (SDS) reducing polyacrylamide gels. Proteins were transferred onto nitrocellulose membranes (Millipore, Billerica, MA) and blocked with 5% skim-milk in PBS for 1 h. After blocking, membranes were incubated overnight at 4°C with primary antibodies to analyze the proteoglycans that are cleaved by ADAMTS activity during the injury phase* in vivo*: anti-aggrecan NITEGE (Abcam, cat. no. ab3775), a monoclonal antibody raised to the C-terminal neoepitope NITEGE (mouse, clone BC-13), which recognizes the aggrecanase generated C-terminal neoepitope EGE373↓374A within the interglobular domain of cleaved aggrecan [8]; anti-versican DPEAAE (Abcam, cat. no. ab19345), a polyclonal antibody raised against the C-terminal neoepitope DPEAAE, which recognizes the aggrecanase generated C-terminal neoepitope DPEAAE^441^↓A^442^ at the N-terminus of V0/V1 versican [18]; anti-brevican (Santa Cruz, cat no. sc-20555), a goat polyclonal antibody raised against a peptide mapped to the N-terminus of human brevican that recognizes full length brevican and ADAMTS aggrecanase generated C-terminal cleavage products [19]. Moreover, we next analyzed ADAMTS1 (Abcam, ab. 28284), raised to an epitope at the amino terminal end of ADAMTS1 after the second furin cleavage site in its propeptide, which detects the active form and autocatalytically processed ADAMTS1 [20]; ADAMTS9 (Abcam, ab. 28279 and it was a* kind gift from Professor Suneel Apte*) raised to the amino terminal end of ADAMTS9 previously described as detecting full-length ADAMTS9 [21]; and ADAMTS15 (Abcam, ab. 28516), raised to the cysteine-rich domain of ADAMTS15 [22]. Secondary antibodies conjugated with horseradish peroxidase followed primary antibody incubations. Enhanced chemiluminescent (ECL) substrate from Biorad (USA) was used for visualization of protein bands. Anti-*β*-actin antibody (Abcam) was used as a loading control.

### 2.3. Immunohistochemistry

Immunostaining was used to evaluate the astrocyte response to SCI. Spinal cord tissue was fixed with 4% paraformaldehyde for 20 min at room temperature, permeabilized for 10 min with 0.1% Triton X-100 in PBS, blocked with 4% skim milk (Thermo), and then incubated for 2 h at room temperature with anti-glial fibrillary acidic protein (GFAP) antibody (1 : 400; DakoCytomation, Carpinteria, CA). The tissue sections were washed in PBS Triton X-100 followed by incubation with goat anti-rabbit antibody conjugated to Alexa 488 fluorophore (1 : 400; Molecular Probes, Invitrogen). Nuclei were counterstained with Hoechst for visualization. Images were captured on Olympus BX51 microscope (Olympus Corporation).

## 3. Results and Discussion

### 3.1. Aggrecan Processing during SCI

**Figure 1:**
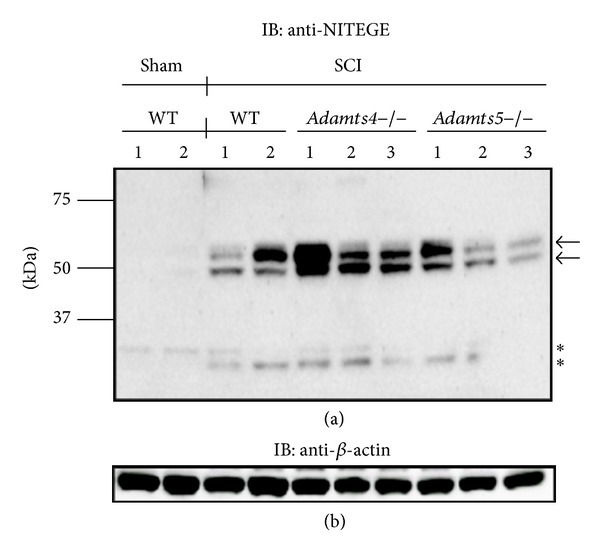
Aggrecan cleavage in* Adamts4*−/− and* Adamts5*−/− mice following SCI. Aggrecan fragments (~50 and 60 kDa) detected with the anti-NITEGE antibody are seen in wild-type,* Adamts4*−/−, and* Adamts5*−/− mice 1 day after SCI (arrows, top panel). Lower molecular weight species (~25 kDa) are also seen in most groups (asterisks, top panel). No aggrecanase activity was detected in sham-operated mice. *β*-Actin was used as a loading control (bottom panel).

Since we previously showed significant upregulation of aggrecan mRNA at 1 day post injury (dpi) [5], we examined aggrecanolysis at this time point. Following SCI, we observed significant ADAMTS generated aggrecan cleavage by Western blot analysis in wild-type,* Adamts4*−/−, and* Adamts5*−/− mice (Figure 1). A predominant ~60 kDa band, representing the G1-NITEGE N-terminal aggrecan fragment generated by ADAMTS proteoglycanases, with an accompanying ~50 kDa band that was readily detectable in all cases except in the sham group (Figure 1), clearly indicating that SCI was responsible for the induction of aggrecan proteolysis. In addition, lower molecular weight bands of ~25 to ~30 kDa were also differentially observed between the sham and SCI groups (Figure 1). These data suggested that ADAMTS4, ADAMTS5, or the other 5 members of the ADAMTS aggrecanase family (ADAMTS1, ADAMTS8, ADAMTS9, ADAMTS15, and ADAMTS20) might cooperate in aggrecan cleavage during spinal cord injury.

### 3.2. Versican Processing during SCI

**Figure 2:**
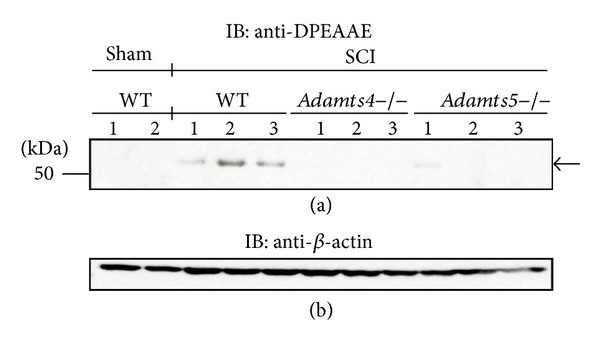
Versican cleavage in* Adamts4*−/− and* Adamts5*−/− mice following SCI. Versican fragments (~70 kDa) detected with the anti-DPEAAE antibody are seen in wild-type but not* Adamts4*−/− and* Adamts5*−/− mice 7 days after SCI (top panel, arrow). No versicanase activity was detected in sham-operated mice. *β*-Actin was used as a loading control (bottom panel).

In contrast to aggrecan, versican mRNA is significantly upregulated at 7 dpi [5]. Western blot analysis on spinal cord protein lysate from 7 dpi revealed the expected ADAMTS proteoglycanase derived 70 kDa versican fragment representing the G1-DPEAAE N-terminal region of V1 versican in the wild-type group (Figure 2). However, in contrast to aggrecan at 1 dpi, little to no G1-DPEAAE fragments were observed in either case of ADAMTS4 or ADAMTS5 knockout mice that had undergone SCI. These data highlight the cooperative nature of the ADAMTS proteoglycanase family to maintain a threshold of versican cleavage during SCI, whereby neither ADAMTS4 nor ADAMTS5, or the remaining ADAMTS proteoglycanases, were able to collectively maintain threshold versican cleavage in the absence of either ADAMTS4 or ADAMTS5. In addition, we also observed an uncharacterized ~37 kDa band using the anti-DPEAAE antibody (see Figure 1 in supplementary materials available online at http://dx.doi.org/10.1155/2014/693746) in all experimental groups.

### 3.3. Brevican Processing during SCI

**Figure 3:**
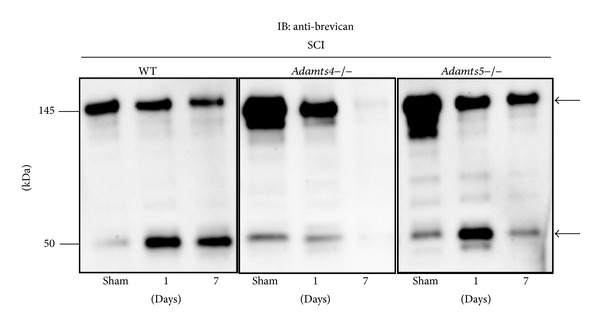
Brevican cleavage in* Adamts4*−/− and* Adamts5*−/− mice following SCI. Full-length brevican (~145 kDa) and brevican fragments (~50 kDa) detected with the anti-brevican antibody are seen in wild-type,* Adamts4*−/−, and* Adamts5*−/− mice 1 and 7 days after SCI (arrows). All groups showed diminished brevican cleavage at 7 days after injury. Minimal brevicanase activity was detected in sham-operated mice.

Western blot analysis to investigate brevican processing was undertaken at both 1 dpi and 7 dpi. Total brevican (~145 kDa) and the ADAMTS proteoglycanase generated 50 kDa brevican fragments were observed at days 1 and 7 in wild-type and* Adamts5*−/− mice (Figure 3), whilst* Adamts4*−/− mice showed total brevican and the ADAMTS-generated brevican fragment (G1-AVESE) [19] at 1 dpi and to a lesser extent total brevican and its fragment at 7 dpi (Figure 3). Brevican cleavage was decreased at day 7 in all groups (Figure 3).

### 3.4. Other ADAMTS Proteoglycanases during SCI

**Figure 4:**
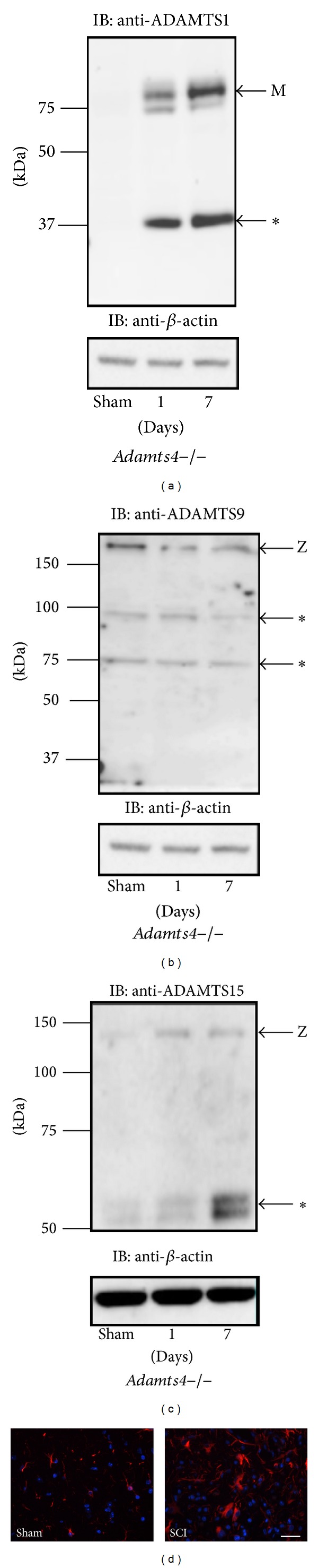
ADAMTS proteoglycanases are expressed during SCI. (a) ADAMTS1 is expressed in spinal cord tissue of* Adamts4*−/− mice 1 and 7 days after injury, m = mature, asterisk = autocatalytic fragment. (b) ADAMTS9 is expressed in spinal cord tissue of* Adamts4*−/− mice in the sham-operated group 1 and 7 days after injury, z = zymogen (inactive), asterisks = uncharacterized fragments. (c) ADAMTS15 is expressed in spinal cord tissue of* Adamts4*−/− mice in the sham-operated group 1 and 7 days after injury, z = zymogen (inactive), asterisk = uncharacterized fragments. In all cases *β*-actin was used as a loading control (bottom panels). (d) Spinal cord injury triggered astrocyte accumulation (reactive astrocytes) at the injury site compared with sham-operated mice. Red = astrocyte, blue = nucleus. Scale bar = 50 *μ*m.

In the absence of ADAMTS4 + ADAMTS5 double knockout mice, we could not rule out the contribution to aggrecan and brevican proteolysis by each other or additional ADAMTS proteoglycanases. However, since aggrecanase activity was clearly present in both ADAMTS4 and ADAMTS5 knockout mice, we used* Adamts4*−/− mice to confirm the presence of a subset of other ADAMTS proteoglycanases during spinal cord injury. Western blot analysis using antibodies specific to a subset of ADAMTS proteoglycanases showed the presence of ADAMTS1, ADAMTS9, and ADAMTS15 in spinal cord tissue (Figures 4(a), 4(b), and 4(c), resp.). An 85 kDa band representing the active form of ADAMTS1 [23] as well as a smaller ~35 kDa fragment representing autocatalysis of ADAMTS1 [23] was observed at days 1 and 7 of spinal cord injury (Figure 4(a)). A ~180 kDa band representing the zymogen (unprocessed) form of ADAMTS9 [24] was observed in all experimental groups (Figure 4(b)), and a ~130 kDa band representing the zymogen (unprocessed) form of ADAMTS15 [22] was also observed in all experimental groups (Figure 4(c)). Lower molecular weight bands were also observed in both cases of ADAMTS9 and ADAMTS15 (Figures 4(b) and 4(c), resp.—asterisks), which may represent autocatalysis of those ADAMTS proteoglycanases [22, 24]. Although all three ADAMTS proteoglycanases were present in ADAMTS4 knockout mice, the most likely candidate for compensatory aggrecanase activity is ADAMTS1 since it was the only ADAMTS shown in its active form, and it robustly appeared in the case of spinal cord injury but not in the sham-operated group (Figure 4(a)). In addition, immunostaining demonstrated astrocyte accumulation at the injury site compared to normal tissue (Figure 4(d)), which could represent the source of ADAMTS proteoglycanase expression.

Spinal cord injury is a severe and essentially irreversible process characterized by excessive CSPGs accumulation and the enzymatic removal of chondroitin sulfate at the site of SCI can promote a regenerative process. In this current study, we induced SCI in mice and examined the activity of the ADAMTS proteoglycanases, a family of extracellular proteinases that cleave hyalectan substrates relevant to the spinal cord, namely, aggrecan, brevican, and versican, and could therefore be relevant to the regeneration process. We previously demonstrated that* Adamts1*,* Adamts5,* and* Adamts9* mRNA expressions were significantly increased in the same SCI mouse model in wild-type mice and suggested that aggrecan, versican, and brevican degradation may be mediated by these and other ADAMTS proteoglycanases produced by reactive astrocytes after SCI [5]. Here, we extended the previous study to utilize* Adamts4*−/− or* Adamts5*−/− mice and studied the consequence of SCI on proteolytic activity upon three major hyalectans that represent predominate sources of CSPGs in the spinal cord.

Aggrecan cleavage was readily detectable in injured spinal cords of both* Adamts4*−/− and* Adamts5*−/− mice. Since both ADAMTS4 and ADAMTS5 are strong aggrecanases [25, 26], this result was perhaps unsurprising. Although double* Adamts4*−/− and* Adamts5*−/− mice are required to reconcile whether one aggrecanase is compensating for the other in response to SCI, it is apparent from this current study that robust aggrecanase remodeling can occur in the absence of either ADAMTS4 or ADAMTS5. In previous studies examining aggrecan destruction utilizing ADAMTS4 + ADAMTS5 double knockout mice, significant aggrecanolysis has been observed [9, 27] suggesting that other ADAMTS aggrecanases can contribute to aggrecan breakdown in addition to ADAMTS4 and ADAMTS5.

Versican is normally expressed in the mouse brain; its upregulation inhibits axonal regeneration after CNS injury [4]. In this current study, we observed ADAMTS proteoglycanase-specific V1 versican cleavage at DPEAAE^441^↓A^442^ in response to SCI in wild-type mice; however,* Adamts4*−/− or* Adamts5*−/− mice were largely protected. Several cases of severely reduced or absent versican proteolysis in single knockout mice have been reported during development including cardiac morphogenesis in ADAMTS5 knockout mice embryos [13], melanoblast colonization in ADAMTS20 knockout mice [28], and folliculogenesis in ADAMTS1 knockout mice [16]. To this end, we conclude that the absence of either ADAMTS4 or ADAMTS5, two of the strongest versicanases [29], significantly reduced the threshold of V1 versican proteolysis in SCI. ADAMTS proteoglycanases act cooperatively to process versican during several developmental processes in the mouse; ADAMTS5, ADAMTS9, and ADAMTS20 are required for interdigital web regression during limb formation, and ADAMTS9 and ADAMTS20 are required for palatal shelf closure during secondary palate formation [15, 30]. Thus it is not unreasonable to conclude this to be cooperatively required in neuronal tissue that is undergoing regeneration after injury. Although our major focus was the DPEAAE^441^↓A^442^ cleavage site targeted by ADAMTS proteoglycanases in V0/V1 versican, the V2 versican splice variant is the major source of versican in the CNS [31]. However the V2 splice variant does not possess the GAG-*β* domain containing the well-described ADAMTS cleavage site, although it is remodeled by ADAMTS proteoglycanases at an alternate site [31] not examined in detail in this current study.

Brevican, unlike versican and aggregan, is specifically expressed in the CNS. In this study, we showed that proteolytic processing of brevican was similar to that of aggrecan with significant processing occurring in both* Adamts4*−/− and* Adamts5*−/− mice with an interesting caveat that its proteolysis was markedly reduced at 7 dpi in* Adamts5*−/− mice and essentially absent at this time-point in* Adamts4*−/− mice. Both ADAMTS4 and ADAMTS5 have previously been reported to proteolytically process brevican and* Adamts5* was found to be overexpressed in glioblastoma tissue compared to normal brain tissue [32].

We demonstrated the presence of ADAMTS1, ADAMTS9, and ADAMTS15 proteins in spinal cords of ADAMTS4 knockout mice and confirmed the active form of one of those proteoglycanases, ADAMTS1, during spinal cord injury. Proteolysis of aggrecan is exerted by ADAMTS proteases including ADAMTS1 [23], ADAMTS4 [33], ADAMTS5 [34], and ADAMTS8 [35] in cartilage tissue. The ADAMTSs that cleave aggrecan are less studied in CNS injury, and especially little is known in the context of SCI. However, accumulating evidence points out their roles in CNS pathologies. ADAMTS1 deficient mice showed efficient brevican and versican V2 cleavage in frontal cortex [36]; however, in this current study its active form was noticeably absent in the sham groups but specifically expressed during spinal cord injury in ADAMTS4 knockout mice suggesting it to be a major contributor of aggrecanase activity during this process.

We focused this study on ADAMTS4 and ADAMTS5, the major aggrecanases under investigation in cartilage destruction in arthritis. ADAMTS4 was first cloned from human brain [37] and is responsive to several CNS conditions such as beta-amyloid, which induces ADAMTS4 upregulation in rat astrocytes [38].* Adamts4*, along with* Adamts1 *mRNA, was markedly elevated in the hippocampus of rats in response to kainate-induced excitotoxic lesion [39] while* Adamts1* expression also increased in a rat model of middle cerebral artery occlusion, [40]. ADAMTS proteoglycanases may also interact with other pathways; for example, ADAMTS4 can signal through MAP ERK1/2 to promote neurite overgrowth independent of its proteolytic activity [41].

The present study was performed in single knockout mice where proteolysis of aggrecan and brevican was indicated. Given that ADAMTS activity likely benefits neurite outgrowth after CNS injury [5, 6], one might hypothesize that abolishing the activity of two or more ADAMTS aggrecanases could adversely affect the recovery process after CNS injury. Although studies in double knockout mice are feasible given that ADAMTS4 + ADAMTS5 knockout mice are reproductively viable, previous studies suggest that additional aggrecan-degrading activity is present in those mice in pathological processes [27]. However, sequentially knocking out additional ADAMTS aggrecanases in mice is not viable due to the embryonic lethality of* Adamts9*−/− mice [30], and* Adamts1 *and* Adamts5 *are separated by only 60 kb on mouse chromosome 16 [42], giving a low probability of segregation of these two* Adamts *genes during meiosis, making it difficult to generate ADAMTS1 + ADAMTS5 knockout mice using standard Mendelian genetic approaches. Taken together, our findings suggest that other members of the ADAMTS proteoglycanase family, such as ADAMTS1, may be responsible for turnover of aggrecan, versican, and brevican cleavage in the mouse spinal cord.

## Supplementary Material

Supplementary figure 1 short description: In addition to the well- characterized 70 kDa band observed with anti-DPEAAE antibody staining that represents the G1-DPEAAE fragment of V1 versican, we also observed an uncharacterized ~37 kDa band in all samples analyzed.
An additional band is associated with versican cleavage in *Adamts4-/-* and *Adamts5-/- *mice: A lower molecular weight ~37 kDa band is observed with the anti-DPEAAE antibody in wildtype and *Adamts4-/-* and *Adamts5-/-* mice 7 days after SCI (top panel - arrow). *β*-actin was used as a loading control (bottom panel).

## Abbreviations

ADAMTS: A disintegrin-like and metalloproteinase domain with thrombospondin-1 motifs
CNS: Central nervous system
CSPG: Chondroitin sulphate proteoglycan
ECM: Extracellular matrix
GAG: Glycosaminoglycan
PG: Proteoglycan
SCI: Spinal cord injury
SDS: Sodium dodecyl sulphate.
