# Supplementary figures and images for: ADAMTS4 and ADAMTS5 Knockout Mice Are Protected from Versican but Not Aggrecan or Brevican Proteolysis during Spinal Cord Injury
Source: Biomed Res Int. 2014 Jul 3;2014:693746. doi: 10.1155/2014/693746 (PMC4101972; doi:10.1155/2014/693746)

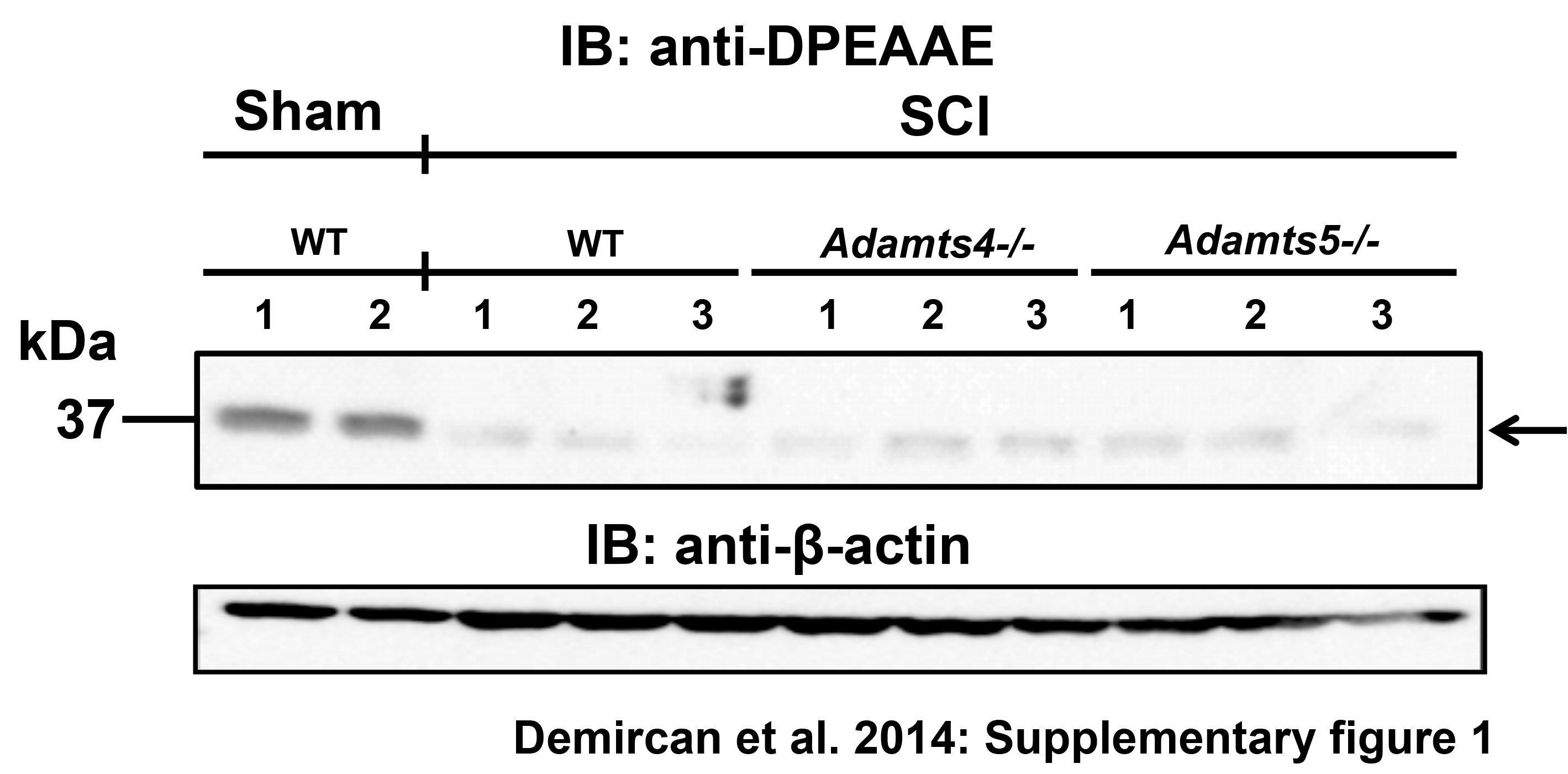

Supplement: Supplementary file 1 — Supplementary figure 1 short description: In addition to the well- characterized 70 kDa band observed with anti-DPEAAE antibody staining that represents the G1-DPEAAE fragment of V1 versican, we also observed an uncharacterized ~37 kDa band in all samples analyzed. An additional band is associated with versican cleavage in Adamts4-/- and Adamts5-/- mice: A lower molecular weight ~37 kDa band is observed with the anti-DPEAAE antibody in wildtype and Adamts4-/- and Adamts5-/- mice 7 days after SCI (top panel - arrow). β-actin was used as a loading control (bottom panel). [file 693746.f1.tif]
